# Optimal Care Pathways for People in Suicidal Crisis Who Interact with First Responders: A Scoping Review

**DOI:** 10.3390/ijerph191811510

**Published:** 2022-09-13

**Authors:** Katelyn Kerr, Ed Heffernan, Jacinta Hawgood, Bronwen Edwards, Carla Meurk

**Affiliations:** 1 Australian Institute for Suicide Research and Prevention, School of Applied Psychology, Griffith University, Brisbane, QLD 4122, Australia; 2 Toowong Private Hospital, Day Programs, Brisbane, QLD 4066, Australia; 3 Savoir Rooms Specialist Medical Practice, Brisbane, QLD 4064, Australia; 4 Queensland Centre for Mental Health Research, Wacol, QLD 4076, Australia; 5 Queensland Forensic Mental Health Service, Brisbane, QLD 4000, Australia; 6 School of Public Health, The University of Queensland, Herston, QLD 4006, Australia; 7 Roses in the Ocean, Brisbane, QLD 4006, Australia

**Keywords:** suicide crisis, suicide prevention, first responders, care pathways

## Abstract

Background: First responders play a vital role in attending to people in suicidal crisis and influencing their care. Aims: To examine existing care pathways and models of care that could be used for people in a suicidal crisis who have come to the attention of first responders. Methods: A scoping review of academic and grey literature published between 2009 and 2019 was conducted, supplemented by consultation with experts, service providers and people with lived experience. Results: The search identified 703 studies. Twenty-three peer reviewed and grey literature articles, as well as one personal communication, were considered eligible for inclusion. Six models, covering 22 programs, were identified. No studies were identified that described care pathways, per se. Co-responder and safe haven models were associated with reduced hospital use and police detentions. Aftercare models were associated with improved well-being and reduction in symptoms. Co-responder, safe haven, and aftercare models were all rated positively by service users. No studies measured the impact on longer term suicidality. Limitations: Inclusion criteria were broad resulting in heterogeneity of studies and designs, limiting comparisons. Few studies employed standardised measurement protocols, reducing the ability to draw sound conclusions. Conclusion: Several novel programs have the potential to support individuals in crisis who encounter first responders.

## 1. Introduction

Suicide is an increasing concern around the world with 700,000 people estimated to die by suicide each year [1]. In Australia, suicide is the 13th leading cause of death, with 12.9 suicides per 100,000 population [2]. Furthermore, it is estimated that for every 100 Australians, three people will attempt suicide in their lifetime, and four in 1000 will attempt suicide in any one year [3]. Additionally, it has been well established that a previous suicide attempt, along with suicidal ideation with intent, are strong predictors of future suicide [4]. One study found 81.8% of index attempt survivors died by suicide within a year [4]. 

Police and paramedics (hereafter ‘first responders’) play a crucial role in helping people through suicidal crises and facilitating appropriate intervention and follow up [5]. Results from a data linkage study in Queensland, Australia, identified that first responders receive in excess of 200 suicide related calls per day, and that over the three-year period from 2014–2017, there was an increase in suicide related calls of approximately 25% across police and ambulance services [6]. 

To date, the predominant care pathway used by first responders in Australia for persons who are in, or at high risk of, a suicidal crisis has been transportation to a hospital emergency department (ED) [7]. However, there is limited, if any, peer-reviewed evidence that transportation to hospital is an optimal response. Recent reports have highlighted significant barriers to care that await those presenting to Australian EDs with mental health problems, including suicide crises [8,9,10]. At the same time, individuals with lived experience of suicide are increasingly vocalising the view that EDs are inappropriate therapeutic environments [11]. In the absence of viable alternatives, or in a situation where an individual in crisis has also committed an offence, they may be transported for detention in a watch house. 

Tailored, effective interventions that can be delivered during or immediately following the first response are vital to support a person out of a crisis and prevent future suicide attempts. In Australia, first responders currently have limited options available to them to assist individuals in crisis, especially after hours, and are further limited by the catchment area the person lives in. 

This scoping review investigates options that are, or could be, available to first responders to help care for a person in suicidal crisis. The paper defines care pathways as the sequential contacts that people in a suicidal crisis have or could have with first responders and care providers [7]. 

### Aims

The current review had three aims:

1. Examine the existing care pathways for people in a suicidal crisis who have come to the attention of first responders;
2. Examine programs which could be used by first responders for suicide prevention;
3. Evaluate the outcome evidence for these programs.

A suicidal crisis was defined as documented instances or accounts of suicidal ideation, voiced risk of suicide or self-harm, intentional self-harm, suicidal behaviours and suicide attempts [6]. Models refer to the overarching care type that is provided (e.g., co-responder; aftercare etc.) while programs refer to the specific, individual services that provide the interventions under the overarching model (e.g., West Moreton Co-Responder; The Way Back Support Service—an aftercare model, etc.)

## 2. Method

### 2.1. Search Strategy

A database search of peer reviewed literature and grey literature was conducted, supplemented by consultation with known experts and snowballing from the original corpus of information. The literature search was informed by the PRISMA standards for systematic review [12]. 

Academic databases searched were: PUBMED, PsycINFO, and Google Scholar. The search term logic was ‘<crisis terms> AND <model of care terms>. Crisis terms were: suicid* crisis, suicid* care, mental health crisis, suicid* prevention; model of care terms were: police, first responder*, model* of care, co-responder, suicid* sanctuary, safe haven, care pathway*, alternative* to admission. The search was limited to articles published over a ten-year period (2009–2019) in the English language. The grey literature search was guided by clinical knowledge, and consultation with known experts. Peer reviewed or published evaluations were sought for each of the identified models, including making direct contact with service providers to uncover published or unpublished materials.

### 2.2. Inclusion Criteria

Initial scoping revealed limited literature on care pathways per se. Therefore, a conceptual framework was developed that focused on identifying literature with respect to the following seven domains: (1) ‘doing nothing’; (2) co-responder models; (3) alternatives to ED presentations; (4) involuntary or voluntary transportation to EDs; (5) safe havens; (6) transportation to the watch-house; and (7) field based brief interventions. This framework was refined iteratively. Models were included in this review if they met the following criteria:

1. Developed explicitly for populations in suicide or mental health crises who come in contact with first responders; OR
2. Judged by the authors to share important similar features with other models that met criteria 1; OR
3. Judged by the authors to be a model which could be utilised by first responders in the future.

Due to the limited evidence-base, inclusion was based on relevance to the aims of the review. No exclusion criteria were set in relation to study type or availability of a formal published evaluation. No quality appraisal of evidence was undertaken as a basis for inclusion or exclusion of models or literature presented. 

### 2.3. Screening and Data Extraction

Documents were screened for inclusion/exclusion through title and abstract screening (title and executive summary screening for reports). For all documents included, the following information was extracted: Reference details; model; description of program (including name, type, and staffing); setting (location); consumer characteristics (age, gender, and characteristics of presentation); evaluation type (if conducted); period of evaluation; sample characteristics (number of cases/individuals); outcome measures; and key outcomes reported.

### 2.4. Analysis

References were summarised individually and then synthesized into the specific categories. 

## 3. Results

**Figure 1:**
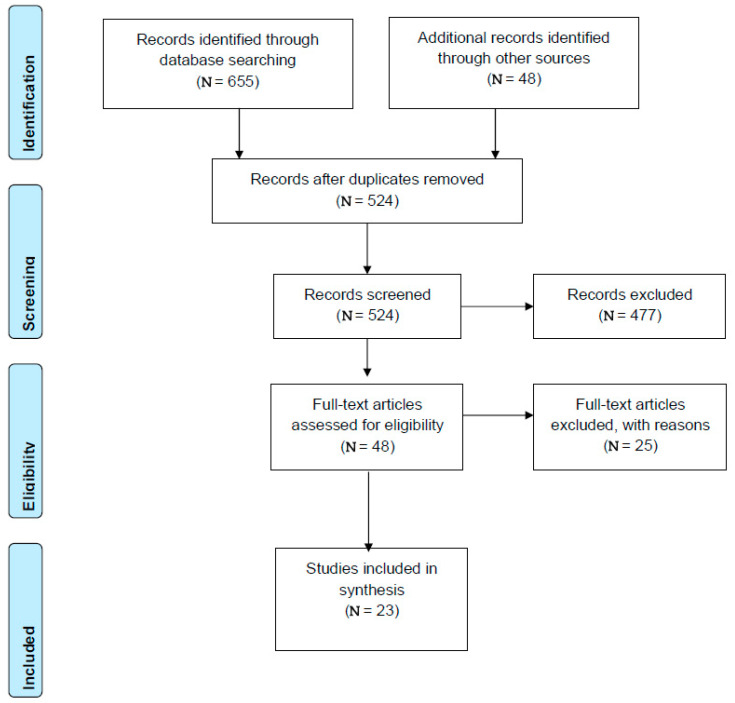
PRISMA flow diagram. Adapted from Moher et al. 2009 [12].

A total of 703 documents were identified. Twenty-three peer reviewed and grey literature articles, spanning six models, met the criteria for inclusion (see Figure 1), and one program whereby information was gathered via personal communication (not included in the PRISMA diagram). Two systematic reviews, 16 evaluated programs, and six programs that do not yet have formal evaluations published were included in the results. These are presented in Table 1.

### 3.1. Models of Care

Six models, covering 22 programs, were identified in the literature and are described below.

Model 1: Co-responder: N = 9. A health clinician co-responds with the police or ambulance or can be contacted immediately for advice. The co-responder conducts mobile assessments, and/or takes over the care of a person in suicidal crisis, allowing first responder crews to return to on-road duties. 

Model 2: Brief contact interventions: N = 1. A time limited and structured intervention with a compassionate response [22].

Model 3: Safe havens (short stays): N = 4. Characterised by safe, comfortable spaces as an alternative to ED.

Model 4: Blended models: N = 1. Holistic, multi-factorial service, encompassing high tech call centres, 24/7 mobile crisis teams, and crisis stabilisation retreats/programs [29].

Model 5: Culturally appropriate crisis responses: N = 2, related to Aboriginal and Torres Strait Islander programs.

Model 6: Aftercare: N = 5. Post-ED programs that provide follow up. Engagement is time limited and linkages to other services is conducted. There exists the possibility of modifying these programs to directly link first responders with aftercare programs. 

### 3.2. Service User Characteristics 

#### Demographics

*Gender.* N = 8 studies. In the brief contact intervention [22] and aftercare models, females were the predominant clientele accounting for 53–64% of clients [32,33,34]. Males were the predominant clientele in the co-responder model and ranged from 51–60% male in individual studies reviewed [17,18,19] and 47–77% male in a systematic review of co-responder models [13]. 

*Age.* N = 9. One co-responder program reported young adults between the ages of 18–29 represented the group most in contact with the program [15], and another found that 51% of clients were aged between 20–39 years of age [19]. Three of the four safe havens stated the minimum age of clients was 18 years [23,24,25]. One safe haven reported the age range of program users as 20–59 [28]. Aftercare models reported on greater age variability, with ages ranging between 15–75 (78% were under 45) [32], 16–80 (m = 31.6) [33], and 18–74 (m = 38.1) [34].

*Cultural Background.* N = 3 studies reported on ethnicity, with two focusing solely on providing support for Aboriginal and/or Torres Strait Islander populations [30,31] and one aftercare program reporting that 14% of their clients identified as Aboriginal and/or Torres Strait Islander [32].

*Referral Reasons.* N = 9 studies reported on reasons for referral. In the co-responder model, severe voiced suicidality, suicide attempts, and crisis as well as severe mental illness were the predominant referral reasons [15,16,17,18,19]. In the brief contact intervention, reasons included low mood, stress, and anxiety, as well as suicidal ideation and self-harm [22]. Safe haven referrals included presentations of stress, anxiety, depression, crisis prevention, and social reasons [23,28]. In the aftercare model, referral was for suicide attempt, self-harm, or suicidal ideation [33].

*Previous Contact with Mental Health Services.* N = 2 studies, both co-responder programs, reported on previous/current contact with mental health services, reporting that 54% [16] and 36% [19] of clients were a registered mental health service client, most known to police [16], and 7% were case managed [19]. Excluding the programs which were designed for clients directly discharging from hospital following a suicide crisis, two studies (one a safe haven and one aftercare program, respectively) reported on hospital use and suicidality. It was found that 67% of clients had attended a hospital emergency department in the past 12 months, with 24% attending three or more times [23], and 51% had a previous reported instance of deliberate self-harm [33].

*Repeat Service Users.* N = 4 studies, all safe havens, commented on repeat service users. They found: 62 individuals made 400 visits [24]; 87 guests made 228 visits [27]; 670 individuals accounted for 4275 attendances at the program [23]; and one noted that the program was visited 56 times which included people who presented multiple times [28].

### 3.3. Outcome Evidence 

*Psychometric Assessments.* N = 4 utilised psychometric assessments or a rating scale system, with all finding improvements in mental health and wellbeing pre to post interventions. See Table 2.

*Reductions in Psychiatric Hospitalisations.* N = 9. Evidence for the co-responder model was conflicting. Puntis et al., 2018 [13] reported a reduction in psychiatric hospitalisations in three studies; however, hospitalisations were found to increase in another three. Rogers et al., 2019 [14] stated the data suggested that co-responder programs resulted in higher hospital admission rates. In contrast, Meehan et al., 2019 [19] found that there was a statistically significant reduction in ED presentations for the six months after the program was introduced and that surveys of police revealed they intended to transport the majority of consumers (82%) to hospital under an Emergency Examination Authority (EEA), but when the co-responders were present, only 23% were transported. These results were supported by two other programs, with both finding a reduction in transportation to EDs [16,18]. Further evidence comes from Coffman et al., 2019 [21], who found a strong reduction in transportation to hospital, instead utilising mental health crisis centres.

In regard to the safe haven model, the limited evidence available suggested that these were beneficial in reducing ED attendances. Of those who had previously attended an ED, 53% showed a decrease in ED attendance following introduction to their program, 19% showed no change and 28% showed an increase in their attendance at an ED [23]. Nearly 30% of those surveyed reported that they would have presented at an ED if safe haven was not there [23]. At another safe haven, 37% of consumers identified that they would have presented to ED if the haven was not open [24]. This equated to 151 ED attendances avoided, approximately 30 per month [24]. However, further analysis of hospital presentation data in the six-months prior and the same six-month time period in 2017 (May to September) revealed that 12% of the safe haven attendees would have presented to ED if they were not open, equating to 10 visits per month, lower than that reported by the attendees themselves [24]. Heyland et al., 2013 [27] reported an estimated 93% deflection rate from ED.

*Reductions in Police Detentions.* N = 3. The co-responder model was associated with a reduction in formal police detentions generally [14], when the co-responder assessment was in-person rather than over the telephone [13]. One safe haven found that police detentions in the wider area in which the program was located reduced, despite a national trend increase [23]. 

*Suicide Prevention/Prevention of Future Suicidal Crises.* Despite the main priority for all the programs’ being to prevent suicide, only N = 1 measured their program’s effectiveness on suicide prevention. The results concluded the aftercare program was successful in resolving suicidal crises, based on statistically significant increases in self-esteem; increased protective factors; and significant decreases in depression and negative suicide ideation; however, no long term follow up was conducted [34].

*Cost Effectiveness.* N = 4 studies investigated cost effectiveness, all part of the co-responder model. The outcomes of a systematic review were inconclusive, with one study finding that co-responders reduced costs by 23%, another finding that it reduced policing costs but increased health provider costs, and one study revealing costs increased by less than 1% [13]. Rodgers et al., 2019 [14] concluded there was a lack of evidence on cost-effectiveness of co-responder models. However, two programs found cost-effectiveness of the co-responder model, with the program being less expensive than the comparator site [16], and another saving an estimated AUD $2,197,800 (AUD $1075 per patient) by transporting clients with mental health issues to a crisis stabilisation centre rather than a hospital emergency department [21].

*Perceptions of Those Utilising the Service.* N = 4. Consumer satisfaction was rated positively. In the co-responder model, consumers commented on reduced waiting times, more flexibility in care options, less need for transportation to hospital, and better utilisation of resources [16]. Seven studies found that consumers were positive with regards to a co-responder model in comparison to their previous experiences with police, but three studies reported clients were dissatisfied with the lack of follow up and referral pathways [13]. For the safe haven model, 85% of consumers stated the program had prevented their crisis, 89% agreed it had helped them manage difficulties at that time, 94% endorsed it had provided a safe place to be, and 18% stated it helped them stay alive [23]. In the aftercare model, one program evaluated this variable finding all respondents (N = 6) being highly satisfied [32].

*Stakeholder Perceptions*. N = 4. Respondents included police [16,17,23], ambulance, mental health staff [16], and staff of the program [17,23] with most being positive. Lee et al., 2015 [17] found that police were more positive about the co-responder program than the clinicians in their ratings of different components of the program. When asked if the co-responder should continue, 100% (N = 65) police officers responded “yes,” with 40% of the clinicians (N = 10) agreeing. In a systematic review of the co-responder model, nine studies found service providers viewed the programs positively and police viewed it neutrally, with the main limitations being availability of the co-responder and restricted hours [13]. 

## 4. Discussion

To the authors knowledge, this is the first scoping review to examine care pathways for people who are experiencing a suicidal crisis that results in contact with first responders. The aims of the review were to examine current care pathways for people in suicidal crisis or identify programs which could be used by first responders as a care pathway and evaluate the evidence base for these programs.

The scoping review identified several novel programs with the potential to support diversion of individuals in crisis away from ED and towards care options that effectively and compassionately meet their needs. Further, existing infrastructure that could be used by first responders to support those in crisis was identified. For example, the potential for transportation of the person to a safe haven instead of an ED, particularly where medical treatment is not indicated. 

Evidence was also found for an important gender variation in consumer access and utilisation across models. Specifically, females were more likely to be clients of brief intervention and aftercare models, while males were the predominant clientele for co-responder models. This may reflect the fact that males come to the attention of police more often [36]. Further variation in consumer utilisation was identified for age groups. Co-responder programs most often serviced young to middle aged adults, while the aftercare programs were utilised by the widest age range. Importantly, service users and stakeholders rated the co-responder and safe haven models positively. 

In regard to evaluation of outcomes, this review found very few scientifically rigorous studies purposely designed to measure the impacts of their intervention, with little generalisation beyond the context and setting they operated in. There was a lack of utilisation of psychometric and other assessments, including mood, mental health, psycho-social and/or quality of life data, to ascertain impacts of the programs; nor was there evidence of longer-term follow up of clients or control and/or comparison group designs. Interestingly, despite the main aim of all programs in this review being to reduce suicidality (ideation and behaviours), there was a lack of appropriately designed research to determine immediate, short- and long-term effectiveness across all models on this outcome. 

Unfortunately, limited synthesis could be provided regarding brief contact interventions (N = 1), blended models (N = 1), and culturally appropriate crisis responses (N = 2), as the programs in these models are awaiting formal evaluation. Whilst a report was produced describing one of the culturally appropriate crisis response services [30], this did not formally measure outcomes associated with the service. 

With regards to specific outcomes, some evidence revealed that safe havens and co-responder models resulted in reduced attendance at EDs and police detentions. These outcomes held true during the hours of operation of the programs, hence providing some evidence to trial extending the hours of operation. According to Coffman et al., 2019 [21], it was also useful to have crisis centres or psychiatric facilities available as alternatives to ED for the co-responders to transport the person in crisis to. Concerning ED presentations, it is noteworthy that no studies explored whether reduced presentations to ED, as a result of accessing the safe haven or co-responder model, reflected positive or negative outcomes. No evidence could be found of the outcomes associated with first responders transporting the person in crisis to hospital nor of police presenting the person to the watch house. Remarkably, since the assertion by Wilhelm et al., 2007 [33] noting the absence of national guidelines for care pathways to facilitate the diversion of people in a suicidal crisis from EDs, there remains no evidence of these to date.

## 5. Limitations

The current review highlighted several methodological limitations of the studies sourced. These include heterogeneity in study design, lack of outcome measurement appropriate to reported aims, overall lack of rigor in evaluation design, and lack of inclusion and limited services for specific subgroups (e.g., people aged 18 years or younger, those living rurally or remotely, people from different cultural backgrounds, and LGBTQIA + populations). Further, there are known restrictions in researching suicide and prevention, given the low base rate of suicide, difficulties recruiting a large enough sample size to determine significant impacts over time, and the expense/costs of conducting longitudinal research. 

Given the clinical importance of identifying appropriate care pathways for people in a suicidal crisis, some programs reported in the grey literature, which have not yet been evaluated were included in this review. Similarly, it is important to note that the analysis of some models of care was derived from a small sample of literature, with some models having only one program included (brief contact interventions and blended models). The search strategy excluded articles of non-English language, which may have resulted in missing programs. 

## 6. Conclusions

The current paper identified six models, covering 22 programs, that provided care to people in suicidal crisis. Co-responder and safe haven models were associated with reduced hospital use and police detentions. Aftercare models that utilised psychological assessments (three of the five programs) showed improved well-being and reduction in symptoms for service users. Co-responder, safe haven and aftercare models were rated positively by service users. Further information is required in order to draw conclusions regarding the effectiveness of brief contact interventions, blended models, and culturally appropriate crisis responses.

Whilst there were limitations in the implementation and measurement of effectiveness, the review also highlighted compassionate responses which service users stated saved their life. Suggestions to expand, modify, or improve some models and programs were also made, such as extending the hours of operation for co-responders and having the ability to utilise crisis centres instead of EDs. Further research is required to determine whether these programs have a positive impact on suicidality (ideation/behaviour) and other outcomes espoused to result from them and to determine which components make a difference to prevent suicidality and suicide in the long term. Such research would be useful for informing future service planning and delivery in the care of those in suicidal crisis.

## Figures and Tables

**Table 1:** Summary of literature.

Author and Year	Evaluation Type	Consumer Characteristics (Age, Gender, and Characteristics of Presentation)	Referral Reasons	Study Period and Sample Size	Outcome Measures Utilised (Yes/No)
**Co-responder: Health clinician co-responds with police or ambulance or can be contacted immediately for advice. Provide advice, conduct mobile assessments, and/or take over the care of a person in suicidal crisis.**					
Puntis et al., 2018 [13].	Systematic review of descriptive and qualitative studies.	Individuals with mental health problems. Males more likely to be referred to the service (range 47–77% of referrals).	N/A	N/A	No.
Rodgers et al., 2019, [14].	Rapid evidence synthesis of systematic reviews, quantitative and qualitative studies.	Individuals with mental illness or in mental health crises.	N/A	N/A	No.
Bouveng et al., 2017 [15].	Descriptive study.	Individuals with severe mental illness or in suicidal crisis. No age restrictions. Age range of cohort seen 5–100 years. Ages 18–29 were the highest service users. 56% female, 43% male, 0.4% unknown. Stockholm, Sweden.	97% deemed high or medium priority. 36% severe suicide threat 25% suspicion of severe psychiatric illness 18% acute crisis 6% severe suicide attempt 3% suspicion of intoxication/overdose 12% other	2015–2016, 12 months. N = 1580 requests for service, data reported on N = 1036 individual.	No.
The Allen Consulting Group, 2012 [16].	Pre-post study with control group.	Individuals in a suicidal or mental health crisis. No age restrictions reported. Gender not reported. Victoria, Australia	48% concern for individual’s welfare 37% section 10 apprehensions 7% family violence 2% assist community mental health team 2% assist ambulance officers 3% other 54% of service users were a registered mental health service client and most were known to police.	2009–2011, 16 months. N = 783 assistance requests for service.	No.
Lee et al., 2015 [17].	Descriptive study (mixed methods).	Individuals in a suicidal or mental health crisis. No age restrictions reported. 60% male, 40% female. Victoria, Australia.	33% threatened suicide 22% welfare concerns 18% psychotic episode 12% assist mental health team or police 7% family violence 3% revoked community order 2% follow up from previous A-PACER involvement	2011–2012, 6 months. N = 296 contacts for service.	No.
McKenna et al., 2015 [18].	Pre-post study (interrupted time series).	Individuals in a mental health crisis. No age restrictions reported. 58.3% male, 41.7% female. Victoria, Australia	22.6% situational crisis including suicidal ideation/threat 21.4% personality disorder 18.5% affective disorder 18.1% psychotic disorder 11.1% no diagnosis 6.6% alcohol and drug affected 0.8% anxiety disorder 0.8% intellectual disability	November 2011–January 2014, 27 months N = 243 people seen.	No.
Meehan et al., 2019 [19].	Pre-post study (interrupted time series).	Individuals with a suicidal or mental health crisis. No age restrictions. 51% aged 20–39 years. 49% Female. West Moreton, Queensland, Australia.	60% threatening suicide/self-harm 22% situational crisis 5% threatening harm to others 13% other 36% of individuals had had previous contact with a mental health service. 7% were currently case managed by a mental health service.	2017, 4 months. N = 171 individuals; N = 226 occasions of service. Direct contact with N = 137.	No.
Heslin et al., 2016 [20].	Pre-post study (interrupted time series) and cost-offset analysis.	Individuals in a mental health crisis. Age and gender not reported. Sussex, England.	Not reported.	For comparison of actual street triage outcomes, 4 month period, June–September, 2014. For overall outcomes, 6 month period before (1 April–30 September, 2013) and 6 month period after implementation (1 April–30 September, 2014) compared, allowing for a settling in period. N = 358 (before period). N = 358 (after period) of which N = 233 were attended by the street triage.	No.
Coffman et al., 2019 [21].	Descriptive study.	Individuals thought to have a mental health problem as identified by a 911 dispatcher or other emergency services. Inclusion = person on an involuntary treatment hold by police and who were uninsured or on Medi-Cal (Santa Clara/Gilroy site); person being on an involuntary treatment hold or those with acute mental health needs who voluntarily consent for services (Stanislaus and Fresno sites). Age and gender not reported. California, United States of America.	Not reported.	September 2015–March 2019, 42 months (3.5 years). N = 2045 people enrolled in the pilots across three sites.	No.
2. **Brief Contact Interventions: Time limited, structured, interventions that aim to deliver a compassionate and proportionate first response to individuals in crisis**					
O’Neill, 2018 [22].	Currently underway.	Individuals in distress who come to the attention of police, ambulance, hospital EDs, or primary care. Adults. Undergoing expansion in stages to include 16 and 17 year olds, and scoping feasibility for those aged 15 years and younger. 57% female, 43% male. Four trial sites across Scotland.	24% self-reported being under the influence of alcohol/substances at point of referral. Presenting problems, reported, included: Stress/anxiety Low mood Suicidal ideation Self-harm	2016–2021, 4.5 years. Interim findings reported on N = 1322 referrals received from 2016 to September, 2018.	No.
3. **Safe Havens (short stays): Characterised by safe, comfortable spaces for people in suicidal or mental health crisis to go as an alternative to presenting to an emergency department.**					
Griffiths and Gale, 2017 [23].	Mixed design. Interrupted time series of impacts, descriptive study of client satisfaction.	Individuals needing mental health support out of hours. Indicates the service is for adults (18 years and over). Gender not reported. Aldershot, Hampshire, United Kingdom.	56% crisis prevention 23% social reason 13% crisis 7% other. Based on a subsample of 92 service users, 67% had attended ED at least once in the previous 12 months and 24% had attended three or more times.	2016–2017, 12 months. N = 4275 attendances at the service. N = 670 individuals.	None.
Price Waterhouse Coopers, 2018 [24].	Cost-benefit analysis.	People seeking mental health support or social connection. Age 18 and over. Gender not reported. Melbourne, Victoria, Australia.	Not reported.	May–September, 2018, 5 month period compared with (1) the same 5 month period 12 months prior and (2) the 6 months immediately prior to implementation of the service. N = 62 individuals made 400 visits to the café during the evaluation period. N = 41 participants completed a survey.	None reported.
N/A Brisbane North Safe Space (My Mental Health, 2018; Wesley Mission Queensland, 2017) [25,26].	No evaluation.	Experiencing or recently experienced psychological distress. 18 years of age or over. Gender not reported. Brisbane, Queensland, Australia.	Not reported.	N/A	None reported.
Heyland et al., 2013. Heyland and Johnson, 2017 [27,28].	Descriptive study.	Individuals in mental health crisis. 18 years of age or over. Age range 20–59. Gender not reported. Chicago, Illinois, United States of America.	62.5% stress + anxiety or depression 25% anxiety 13% depression	2015, 8 weeks (clients followed up 30 days post separation with service) Total N = 228 visits by 87 individuals. Evaluation included N = 56 visits by 16 clients.	Yes.
4. **Blended Models: Provide a holistic, multi-factorial, comprehensive model of service. Crisis Now encompasses high tech call centres, 24/7 mobile crisis teams, and crisis stabilisation retreats/programs.**					
Crisis Now (NAASP, 2016) [29]	No evaluation currently.	N/A	N/A	N/A	N/A
5. **Culturally appropriate crisis responses:** Focused on meeting the needs of specific cultural groups.					
Dudgeon et al., 2017 [30].	Not described.	Aboriginal and Torres Strait Islander individuals, family, kin and community affected by critical incidents, including suicide or high risk of suicide, murders or multiple casualty events that place those known to the deceased at elevated suicide risk. No age restrictions reported. Gender not reported. Four trial sites across Western Australia, Australia.	Not reported.	2015–2016, ~12 months N = 46 Aboriginal Torres Strait Islander families affected by suicide or critical incident.	None.
N/A National Indigenous Crisis Response Service (NICRS); Similar to ATSIPEP (above). Thirrili., 2017 [31].	None currently.	N/A	N/A	N/A	N/A
6. **Aftercare services: Provide follow up support to people who have recently presented to ED with a suicidal crisis. Time-limited engagement with the aim of linkage to services to prevent suicidality in the future. Possibility of modifying these services so that first responders link directly with post-ED services.**					
beyondblue, 2016 [32].	Mixed design. Pre-post study (quantitative and qualitative). Descriptive study of client characteristics.	People recently discharged from hospital for a suicide attempt of suicidal crisis. No age restrictions reported. 78% under 45 years of age. Age range 15–75. 40% male, 60% female. Darwin, Northern Territory, Australia.	Not reported.	2014–2015, 18 months N = 122 referrals, N = 87 individuals who were recently discharged from hospital due to suicide attempt or suicide crisis. N = 46 interviews with stakeholders.	Yes.
N/A Peer Acceptance, Understanding, Support and Empathy (PAUSE); (Schneck, 2019) (Kezia Schneck, Peer Support Worker Brook RED. Personal communication via telephone. 15 January 2019.)	Evaluation currently underway.	People identified as requiring peer support following suicidality. Brisbane, Queensland, Australia.	N/A	N/A	N/A
Wilhelm et al., 2007 [33].	Mixed design. Pre-post study of outcomes, descriptive study of client characteristics and feedback.	People who present to St Vincent’s hospital ED for deliberate self-harm or suicidal ideation. No age restrictions reported. Mean age = 31.6 years. Age range 16–80. 57% female, 43% male. Sydney, New South Wales, Australia	Reasons for ED presentations: 66% overdose 17% suicidal ideation 12% cutting 2% hanging 6% other self-harm methods 51% of attenders had a previous reported instance of deliberate self harm.	1998–2005, 7 years. N = 456 individuals.	Yes.
Surgenor et al., 2015 [34].	Pre-post study.	Individuals in suicidal crisis. No age restrictions reported. Age range 18–74. Mean age 38.1, 44.4% male, 55.6% female. Ireland.	Not reported.	No date range given. N = 432 individuals invited to participate pre-therapy.	Yes.
N/A Veteran Suicide Prevention Pilot; Department of Veterans Affairs, 2018 [35].	No evaluation currently.	Military veterans following discharge from hospital after a suicidal crisis. Australia.	N/A	N/A	N/A

**Table 2:** Assessment.

Model	Author	Assessment	Outcomes
Safe Haven	Heyland and Johnson, 2017, [28].	Subjective Units of Distress Ratings (SUDS; 0 = no distress with 10 = highest distress). Pre and post.	Reduction of approximately 2 points.
Aftercare	beyondblue, 2016 [32].	WHO-5—World Health Organisation Well-Being Index (0 = worst imaginable well-being—25 = best imaginable well-being).	On average a 10 point improvement was noted (from 12.5 to 22.5).
Aftercare	Wilhelm et al., 2007 [33].	1. Centre for Epidemiological Studies Depression Scale (CES-D; score range 0–60) 2. FANTASTIC lifestyle checklist (0–50. Higher score indicates greater control over one’s lifestyle).	1. Mean CES-D on intake (N = 282) was 35.7 (SD = 12.0) with 95% of guests scoring 16 or more, indicating possible depression and 85% scoring 23 or more, indicating significant depression. Statistically significant reduction in scores for participants who completed all three sessions and the post-test (N = 40, m = 17.9, SD = 12.9). Mean FANTASTIC scores 25.9 (SD = 7.3) at intake, no follow up scores available.
Aftercare	Surgenor et al., 2015 [34].	1. Single item indicator (“I have high self-esteem”) rated on 5-point scale (1 = lowest self-esteem; 5 = highest self-esteem) 2. Patient Health Questionnaire (PHQ-9). Lower score indicates less depression. 3. The Positive and Negative Suicide Ideation Inventory (PANSI). Four positive and four negative items were utilised. Lower scores for negative items indicate fewer negative symptoms. Higher scores for positive items (for protective behaviours) indicate greater protective factors.	1. Mean self-esteem score was 1.76 (SD = 1.07) pre-test and 2.79 (SD = 1.08) post-test. 2. Mean PHQ-9 score was 18.58 (SD = 5.77) pre-test and 10.87 (SD = 7.47) post-test. 3. Mean negative suicidal ideation was 13.04 (SD = 4.22) pre-test and 7.77 (SD = 4.82) post-test. Mean positive suicidal ideation was 9.48 (SD = 3.69) pre-test and 13.76 (SD = 3.66) at post-test. Changes in scores pre- and post-treatment were statistically significant (*p* < 0.001) for all measures.

## Data Availability

Not applicable.
